# Macrophage migration inhibitory factor activates the inflammatory response in joint capsule fibroblasts following post-traumatic joint contracture

**DOI:** 10.18632/aging.202505

**Published:** 2021-02-17

**Authors:** Yuxin Zhang, Shenji Lu, Shuai Fan, Lili Xu, Xin Jiang, Kexin Wang, Bin Cai

**Affiliations:** 1 Department of Rehabilitation Medicine, Shanghai Ninth People's Hospital Affiliated to Shanghai Jiao Tong University School of Medicine, Shanghai 200011, China; 2 Shanghai Key Laboratory of Orthopedic Implants, Shanghai Ninth People's Hospital, Shanghai Jiao Tong University School of Medicine, Shanghai 200011, China; 3 School of Kinesiology, Shanghai University of Sport, Shanghai 200438, China

**Keywords:** macrophage migration inhibitory factor, post-traumatic joint contracture, inflammation, fibroblasts, fibrosis

## Abstract

Objectives: Joint capsule fibrosis caused by excessive inflammation leading to post-traumatic joint contracture (PTJC). Fibroblasts trigger inflammation under the challenge of various proinflammatory cytokines. Macrophage migration inhibitory factor (MIF) is a prominent proinflammatory cytokine involved in inflammation- and fibrosis-associated pathophysiology, we investigated the role of MIF in PTJC.

Methods: Using rat PTJC model and fibroblast inflammation model, we detected MIF expression in posterior joint capsule. Primary joint capsule fibroblasts (JFs) were used to investigate the effects of MIF on cell proliferation, migration and proinflammatory cytokines production. The mechanism of JF-mediated events was evaluated by qRT-PCR, western blot and immunoprecipitation. We screened the mRNA expression profile to identify gene candidates that mediate the effect of MIF on JFs.

Results: MIF increased in posterior joint capsule following PTJC and co-localized with fibroblasts. Injection of MIF inhibitor significantly suppressed joint capsule inflammation and fibrosis. *In vitro*, MIF promoted JF proliferation, migration, and inflammation by regulating mitogen-activated protein kinase/nuclear factor-κB pathway through coupling with CD74. Transcriptome analysis revealed that lipid metabolism-related factors Pla2g2a, Angptl4, and Sgpp2, downstream of MIF/CD74, were potentially implicated in JF inflammation.

Conclusion: MIF/CD74 axis elicited JF inflammation and may provide new therapeutic targets for joint capsule fibrosis in PTJC.

## INTRODUCTION

As the most common chronic musculoskeletal complication after trauma or surgery, post-traumatic joint contracture (PTJC) is mainly characterized by the loss of range of motion (ROM) in moveable joints and limited social participation, often leading to lifelong dysfunction [1–3]. Joint capsule fibrosis caused by excessive inflammation, is the central link and pathogenic basis of PTJC [4–7]. Therefore, conservative drug treatments such as non-steroidal anti-inflammatory drugs (NSAIDs) are widely used to relieve inflammation in traumatic joints [2, 4]. However, due to their limited efficacy and inevitable side effects, the application of NSAIDs has been greatly restricted. Consequently, it is necessary to explore novel therapeutic targets for PTJC and develop more effective treatment strategies.

Fibroblasts, traditionally recognized as quiescent cells responsible for extracellular matrix (ECM) synthesis and remodeling, are increasingly appreciated as key players in inflammation and immune regulation during injury [8]. Fibroblasts together with macrophages, neutrophils, mast cells, and other immune cells are the main source of innate immune responses as they synergistically produce inflammatory mediators such as complement, tumor necrosis factor-α (TNF-α), interleukin (IL)-1β, IL-6, and chemokines [8–11]. Fibroblasts constitutively express a series of receptors related to innate immune response [12–14]. Thus, they are highly sensitive to infections or endogenous tissue damage. These events may lead to the amplification and transformation of acute inflammation into chronic inflammation [15]. Blocking inflammatory signaling in fibroblasts during joint trauma may represent an effective treatment strategy for reducing harmful inflammatory stimuli in the joint capsule.

Macrophage migration inhibitory factor (MIF) is a highly potent proinflammatory cytokine, discovered early due to its ability to inhibit the random migration of macrophages [16]. Subsequent studies found that it was ubiquitously expressed by a variety of cells, including T cells, endothelial cells, macrophages, hepatocytes, astrocytes, and fibroblasts [17]. As a key upstream mediator of inflammation, MIF is involved in the development of inflammation-related diseases such as systemic infection, sepsis, autoimmune diseases, and osteoarthritis, through regulating the pathophysiological processes of inflammation, immunity, wound healing, and cell proliferation and migration [18–21]. Consequently, MIF is a potential therapeutic target for these diseases. Knockout of MIF in elderly mice reduced the severity of osteoarthritis [22], and MIF-specific inhibitor treatment exhibited effective anti-inflammatory effects *in vitro* and *in vivo* [23].

MIF exerts its pathophysiological effects by interacting with surface receptor CD74, which forms a receptor complex with CD44 or chemokine receptors CXCR2, CXCR4, and CXCR7 [20, 24, 25]. The rapid phosphorylation of the mitogen-activated protein kinase (MAPK) pathway is an important intracellular signaling event triggered by MIF [17, 26–28]. The MIF/CD74 axis promotes chemokine CCL5 release, in turn attracting M2 macrophages migration to the spinal cord injury site [29], which is closely related to inflammation regression or entry into chronic stage. We previously found that the COX2/PGE2 inflammatory pathway, targeted by NSAIDs, is activated by the MIF/CD74 axis in astrocytes [30]. These evidences imply that MIF has the ability to regulate the inflammatory microenvironment of injured tissues. However, whether JFs respond to MIF-mediated pathological processes remains unknown. Here, we examined MIF expression in rat damaged joint capsules and analyzed the MIF-induced inflammatory signaling in JFs. In addition, potential inflammation-related target molecules downstream of MIF/CD74 axis were also evaluated. Our results indicated that the MIF/CD74 axis exerted its histopathological function by activating JF inflammation during PTJC.

## RESULTS

### MIF was significantly increased in the posterior joint capsule following PTJC

**Figure 1:**
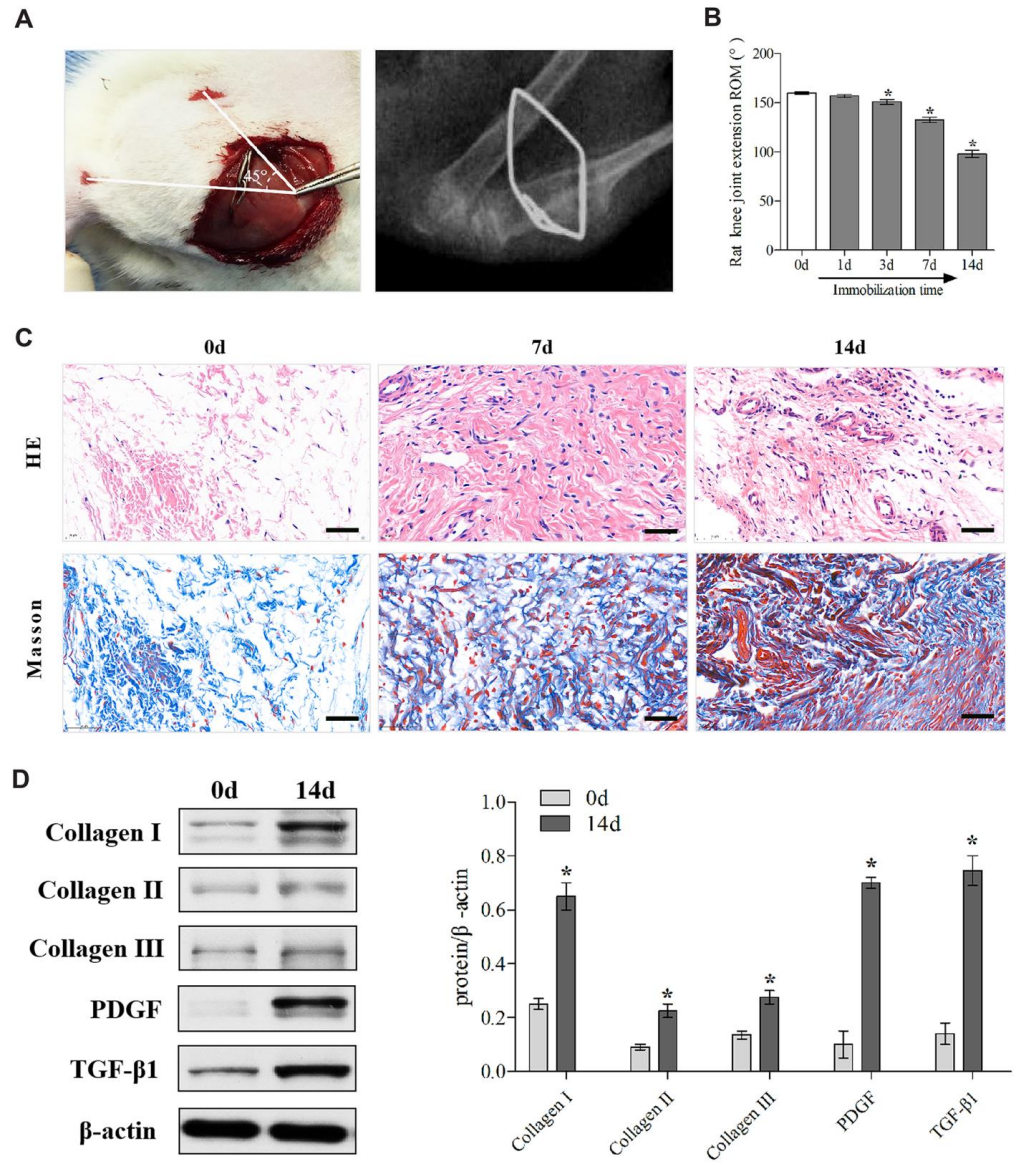
**Establishment and identification of the rat knee joint PTJC model.** (**A**) Schematic and X-ray of rat knee joint post-traumatic immobilization. (**B**) Measurement of extension ROM of the affected knee joint at Day 0, 1, 3, 7, and 14 post-modelling. (**C**) HE and Masson staining of the posterior joint capsule of the affected knee at day 0, 7, and 14 post-modelling. Scale bars, 50 μm. (**D**) Expression of fibrosis-associated protein (collagen I, collagen II, collagen III, PDGF, and TGF-β1) in the posterior joint capsule at day 0 and 14 post-modelling were assessed via western blot. Endogenous β-actin was used as an internal control. Error bars represent standard deviation. *P <0.05 compared with the day 0 group.

**Figure 2:**
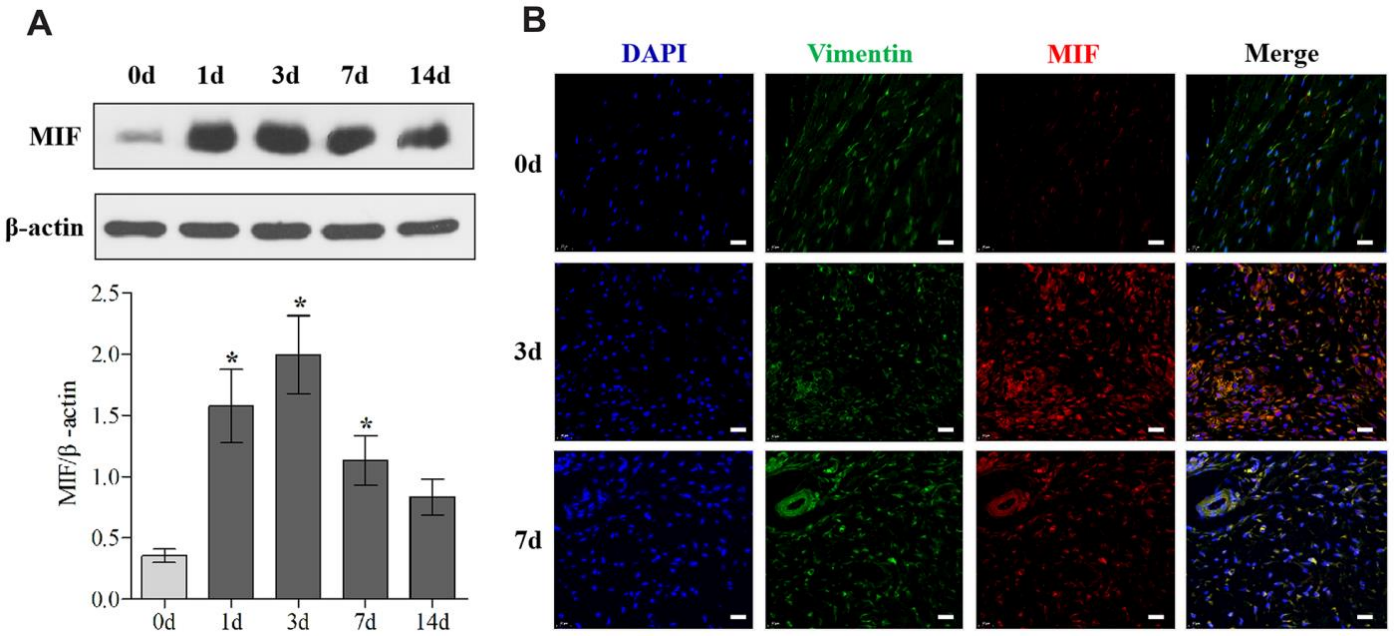
**MIF expression was upregulated in the posterior joint capsule after PTJC.** (**A**) Western blot analysis of MIF in the posterior joint capsule at day 0, 1, 3, 7, and 14 after PTJC induction. Endogenous β-actin was used as an internal control. (**B**) Immunostaining showed colocalization of MIF (red) with JFs in the posterior joint capsule at day 0, 3, and 7 after PTJC induction. Cell nuclei were stained with DAPI (blue). Vimentin was used as a fibroblast marker (green). Scale bars, 20 μm. Error bars represent standard deviation. *P <0.05 compared with the day 0 group.

**Figure 3:**
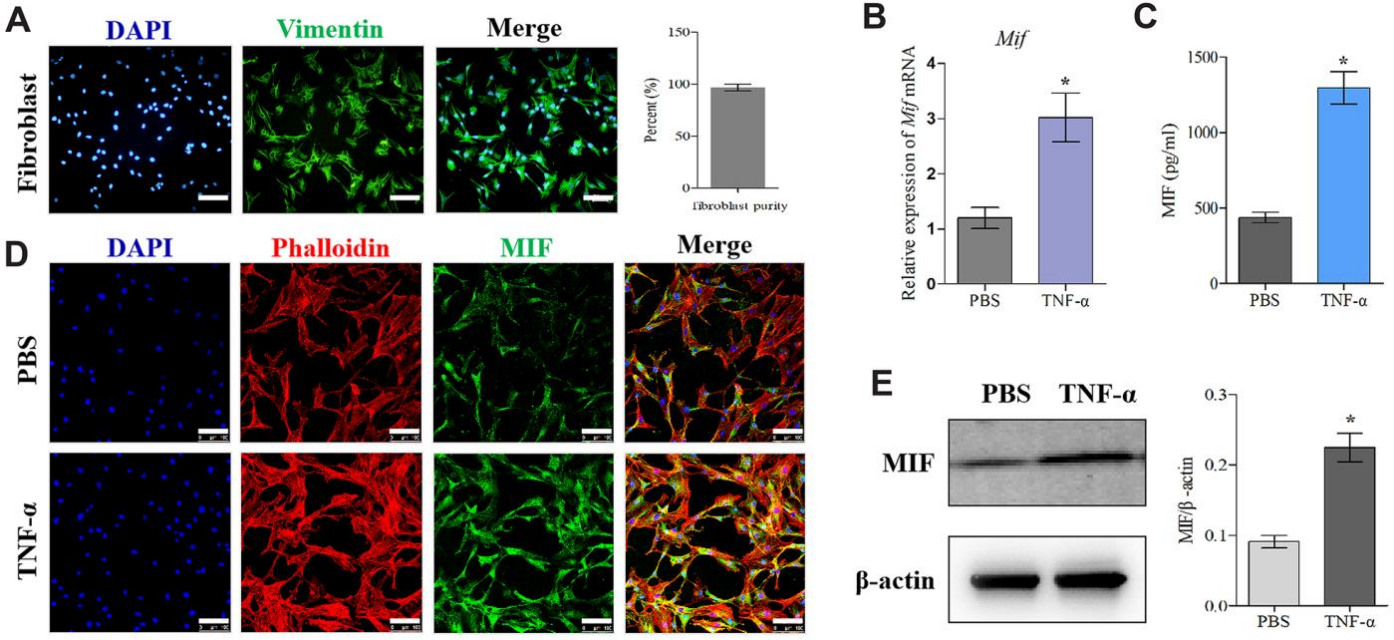
**MIF expression increased in TNF-α-induced primary JF inflammation model.** (**A**) Immunofluorescence of purified primary JFs. Vimentin was used as a marker of JFs (green). Cell nuclei were stained with DAPI (blue). Scale bar, 100 μm. (**B**–**E**) Expression of MIF in JFs in response to 20 ng/mL TNF-α treatment for 24 h was determined via qRT-PCR (**B**), ELISA (**C**), and immunofluorescence (**D**), and western blot (**E**). Phalloidin was used to stain the cytoskeleton (red); cell nuclei were stained with DAPI (blue). Scale bar, 100 μm. Error bars represent standard deviation. *P <0.05 compared with the PBS group.

Firstly, we established the rat PTJC model (Figure 1A) and measured knee extension ROM after myotomies of the trans-articular muscles [31]. The normal knee extension ROM was 160.4 ± 2.5° C, but it reduced to 98.3 ± 3.5° C 14 days post-modelling (Figure 1B). Histological analyses of the posterior joint capsule revealed an increase in inflammatory cell infiltration (HE), collagen fiber hyperplasia and disordered arrangement (Masson) (Figure 1C). Fibrosis-related protein expression was upregulated (Figure 1D). The above changes were similar to those observed in humans and indicated that inflammation and fibrosis were closely related to PTJC. We then examined whether MIF was associated with joint capsule fibrosis. Western blotting revealed that MIF remarkably increased in the posterior joint capsule and reached peak levels on the third day (Figure 2A). Immunostaining showed that MIF co-localized with fibroblasts, the main cellular component of the joint capsule. Consistently, the fluorescence intensity of MIF was markedly enhanced after joint injury, indicating that the production of MIF was synchronous with JF activation (Figure 2B). To further confirm MIF expression in JFs within the post-traumatic inflammatory environment, we cultured primary JFs (Figure 3A) and treated them with TNF-α to establish an *in vitro* inflammatory model [32, 33]. Results revealed that TNF-α potently induced MIF production by JFs (Figure 3B–3E). In general, MIF may thus affect joint capsule inflammation and fibrosis by regulating JF function.

### Inhibition of MIF in the lesion area attenuated inflammation and fibrosis in PTJC

**Figure 4:**
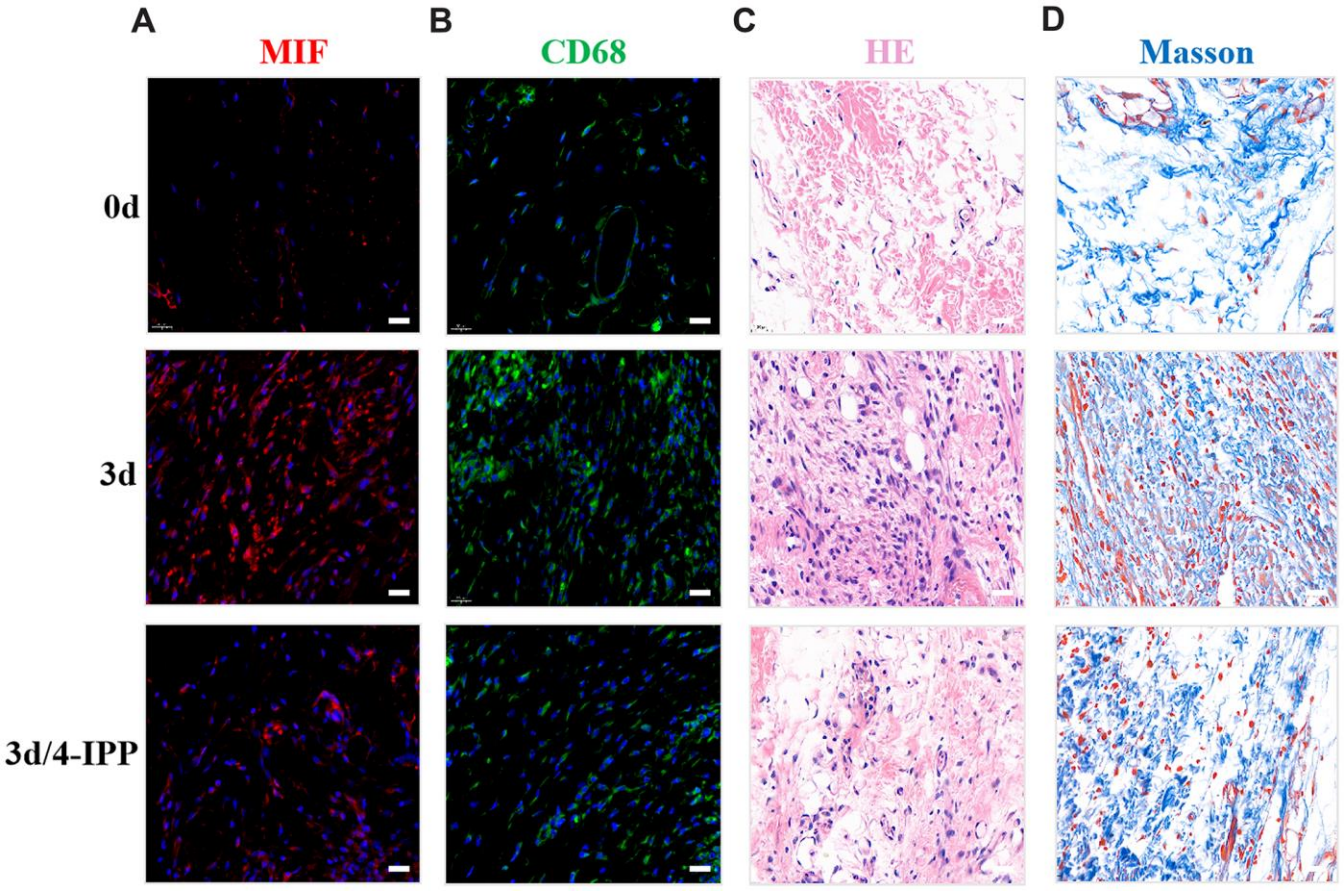
**Inhibition of MIF in the lesion area attenuated posterior joint capsule inflammation and fibrosis.** (**A**) Expression of MIF (red) in the posterior joint capsule was assessed via immunostaining at 0 d, 3 d and 3 d after injection of 4-IPP. (**B**) Immunostaining of CD68-positive macrophages (green) in the posterior joint capsule. (**C**) HE staining of the posterior joint capsule. (**D**) Masson staining of the posterior joint capsule. Scale bars, 20 μm.

To further evaluate the influence of MIF in PTJC, 4-IPP/saline was injected into the lesion site. Three days after injection, we performed histological analysis of posterior joint capsule sections. Immunostaining revealed that 4-IPP injection effectively suppressed MIF (Figure 4A) and CD68-positive macrophages expression (Figure 4B). Similarly, HE staining revealed that inflammatory cell infiltration was profoundly decreased following 4-IPP treatment (Figure 4C). In addition, 4-IPP injection also attenuated collagen fiber hyperplasia as observed by Masson staining (Figure 4D). These data indicated that MIF inhibition had anti-inflammatory and anti-fibrotic effects in PTJC.

### MIF activated inflammatory responses of joint capsule fibroblasts (JFs)

**Figure 5:**
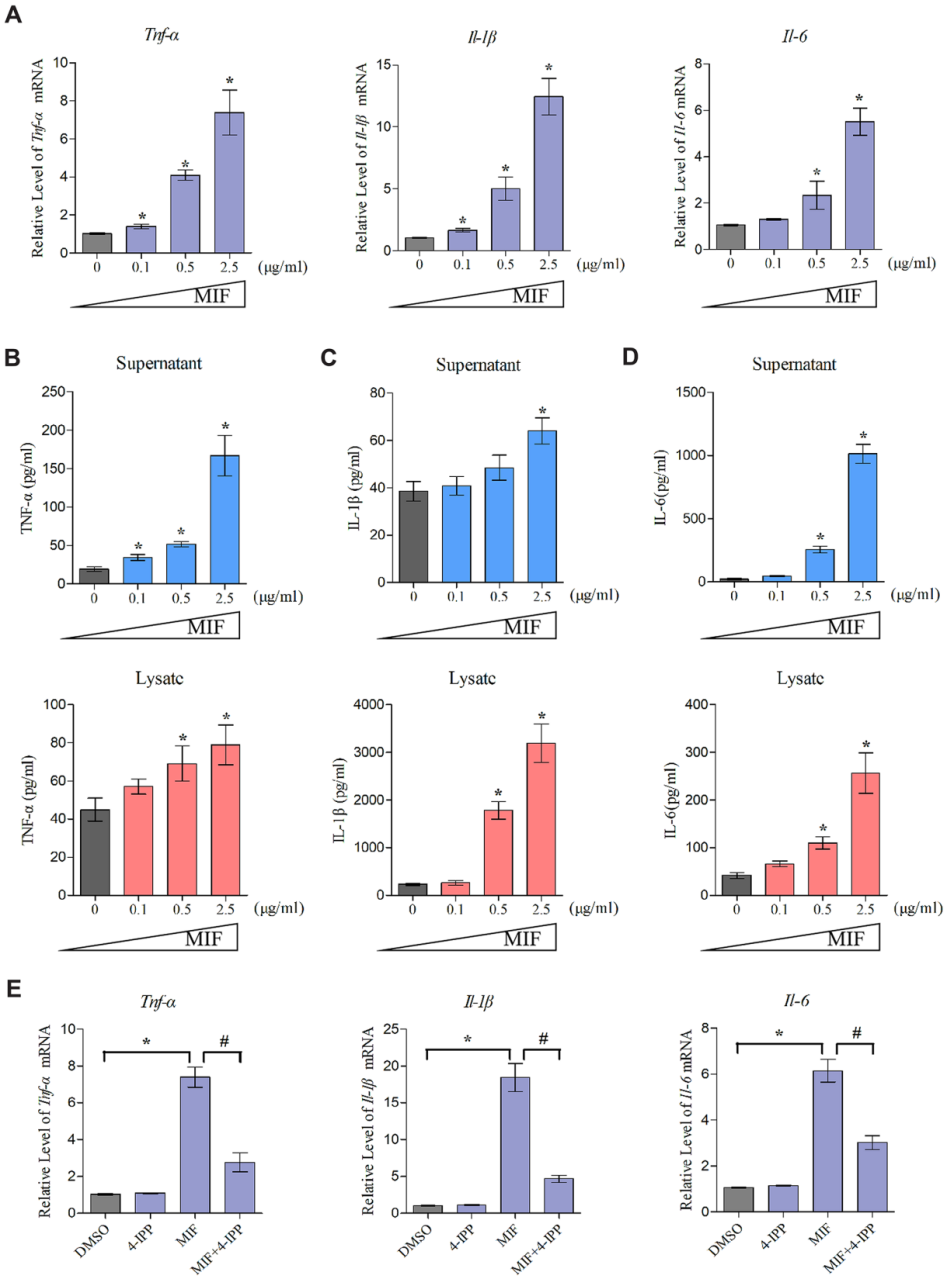
**MIF activated inflammation in JFs.** (**A**) Expression of *Tnf-α*, *Il-1β*, and *Il-6* was assessed via qRT-PCR following JFs treatment with 0–2.5 μg/mL recombinant MIF for 24 h. (**B**–**D**) Levels of TNF-α (**B**), IL-1β (**C**), and IL-6 (**D**) in cell supernatants and lysates were measured using ELISA. (**E**) JFs were treated with 2 μg/mL recombinant MIF combined with 50 μM 4-IPP for 24 h, qRT-PCR evaluated *Tnf-α*, *Il-1β*, and *Il-6* expression. Error bars represent standard deviation. *P <0.05 compared with the 0 μg/mL or DMSO group. #P <0.05 compared with the MIF group.

Extensive studies have emphasized the proinflammatory effects of MIF on various immune and inflammatory cells. To elucidate MIF’s effects on JF inflammation, we detected the expression of proinflammatory cytokines TNF-α, IL-1β, and IL-6 at both transcriptional and translational levels following stimulation with 0–2.5 μg/mL MIF for 24 h. qRT-PCR demonstrated that all cytokines significantly increased in a concentration-dependent manner (Figure 5A). Detection of these cytokine levels in cell supernatants and lysates via ELISA revealed similar changes (Figure 5B–5D). Adding 50 μM 4-IPP to the cultures effectively blocked MIF’s effects (Figure 5E). These data indicated that MIF could activate JF inflammation. Considering that collagen fiber hyperplasia and matrix metalloproteinases (MMPs) production are also involved in the pathological process of joint capsule fibrosis [4–7], we further verify the effects of MIF on JFs. qRT-PCR shown that MMPs (*Mmp-1*, *Mmp-13*) and collagen (*Col1a1*, *Col3a1*) production were also significantly induced by MIF (Supplementary Figure 1).

### MIF facilitated the proliferation and migration of JFs

**Figure 6:**
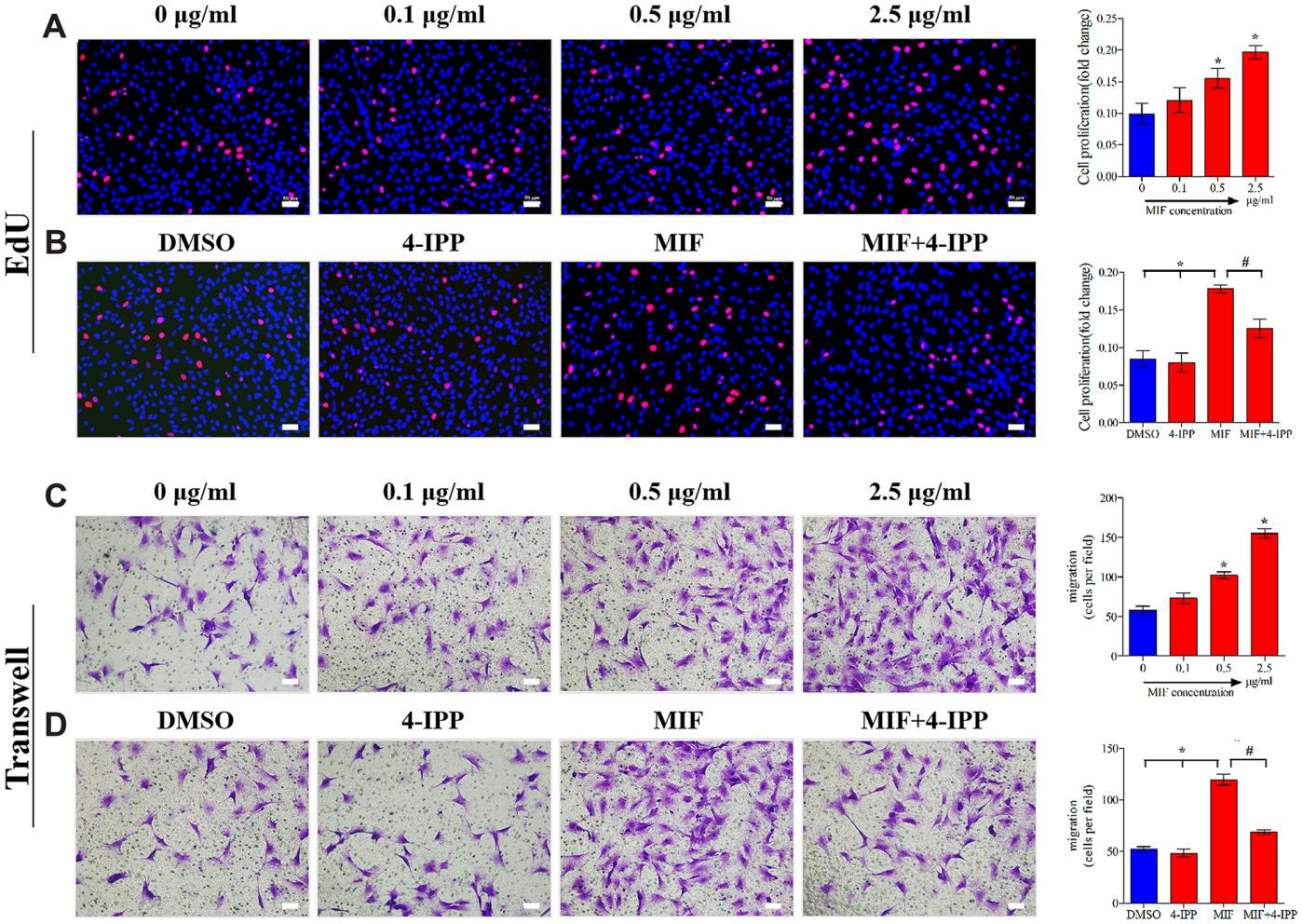
**MIF promoted the proliferation and migration of JFs *in vitro*.** (**A**, **C**) JFs were treated with 0–2.5 μg/mL recombinant MIF for 24 h before testing cell proliferation using an EdU assay (**A**) and cell migration via a Transwell assay (**C**). (**B**, **D**) JFs were treated with 2 μg/mL recombinant MIF combined with 50 μM 4-IPP for 24 h followed by EdU assay (**B**) and Transwell assay (**D**). Scale bar, 50 μm. Error bars represent standard deviation. *P <0.05 compared with the 0 μg/mL or DMSO group. #P <0.05 compared with the MIF group.

Inflammatory and immune responses are the basis of fibrosis, during which fibroblasts activate, proliferate, differentiate, and eventually lead to chronic inflammation through coordination with immune cells [34, 35]. MIF can promote inflammatory and immune cell proliferation and then aggravate inflammatory responses [17, 18, 36]. To further verify the effects of MIF on JFs, EdU (Figure 6A) and Transwell assays (Figure 6C) were performed. We found that MIF at concentrations higher than 0.5 μg/mL effectively promoted JF proliferation and migration, whereas adding 50 μM 4-IPP to MIF-treated cultures significantly attenuated these effects (Figure 6B, 6D). These data indicated that increased MIF in the damaged joint capsule promoted the proliferation and migration of JFs.

### MIF activated MAPK and NF-κB signaling in JFs

**Figure 7:**
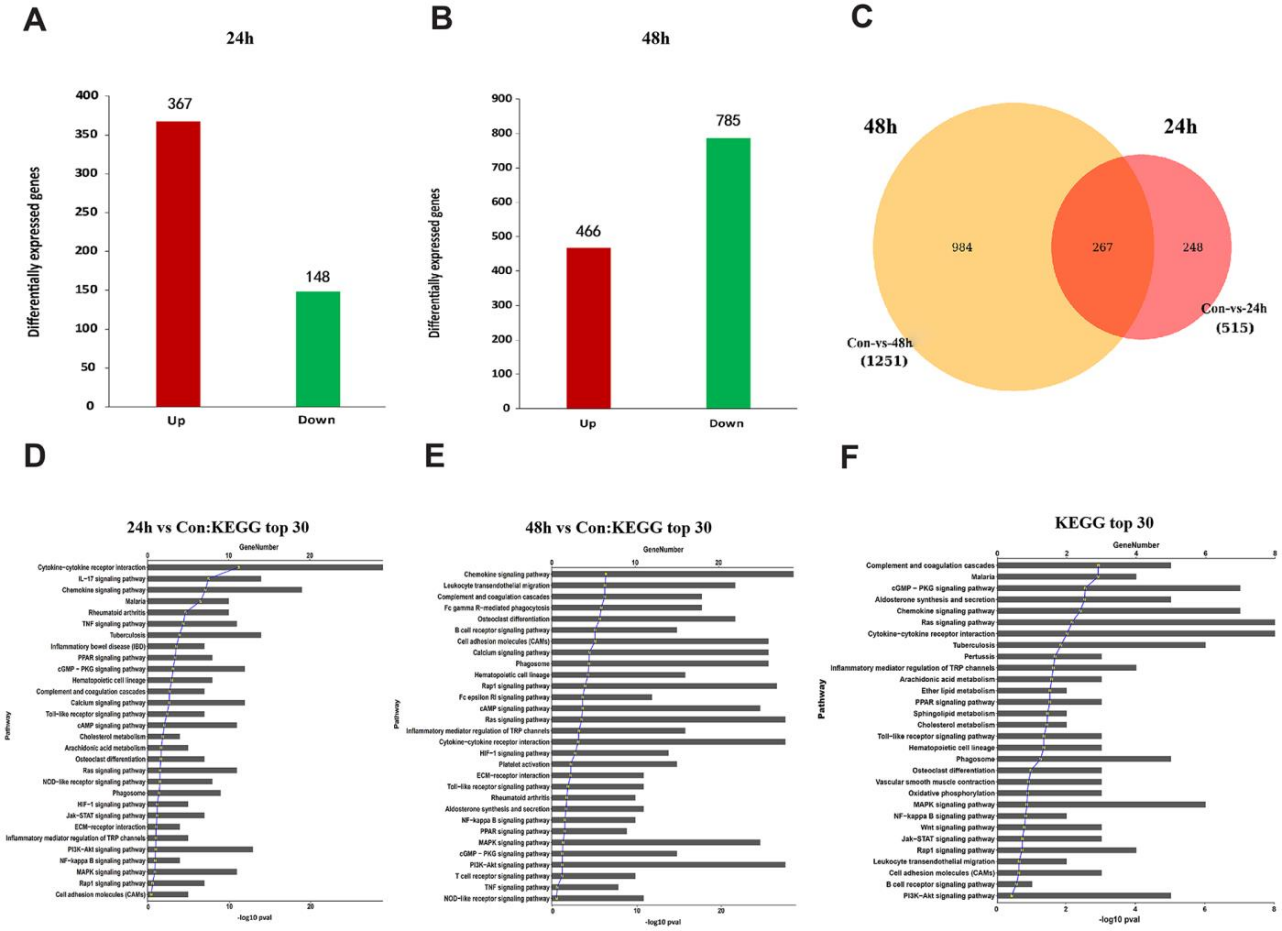
**Functional annotation of DEGs in JFs following treatment with MIF.** (**A**, **B**) Bar graphs of DEGs following treatment with 2 μg/mL recombinant MIF for 24 h (**A**) and 48 h (**B**). (**C**) Integration of DEGs at 24 and 48 h. (**D**, **E**) Top 30 most significantly enriched groups for the DEGs related to pathways following treatment with 2 μg/mL recombinant MIF for 24 h (**D**) and 48 h (**E**). (**F**) Top 30 most significantly enriched groups for integrated DEGs related to pathways.

**Figure 8:**
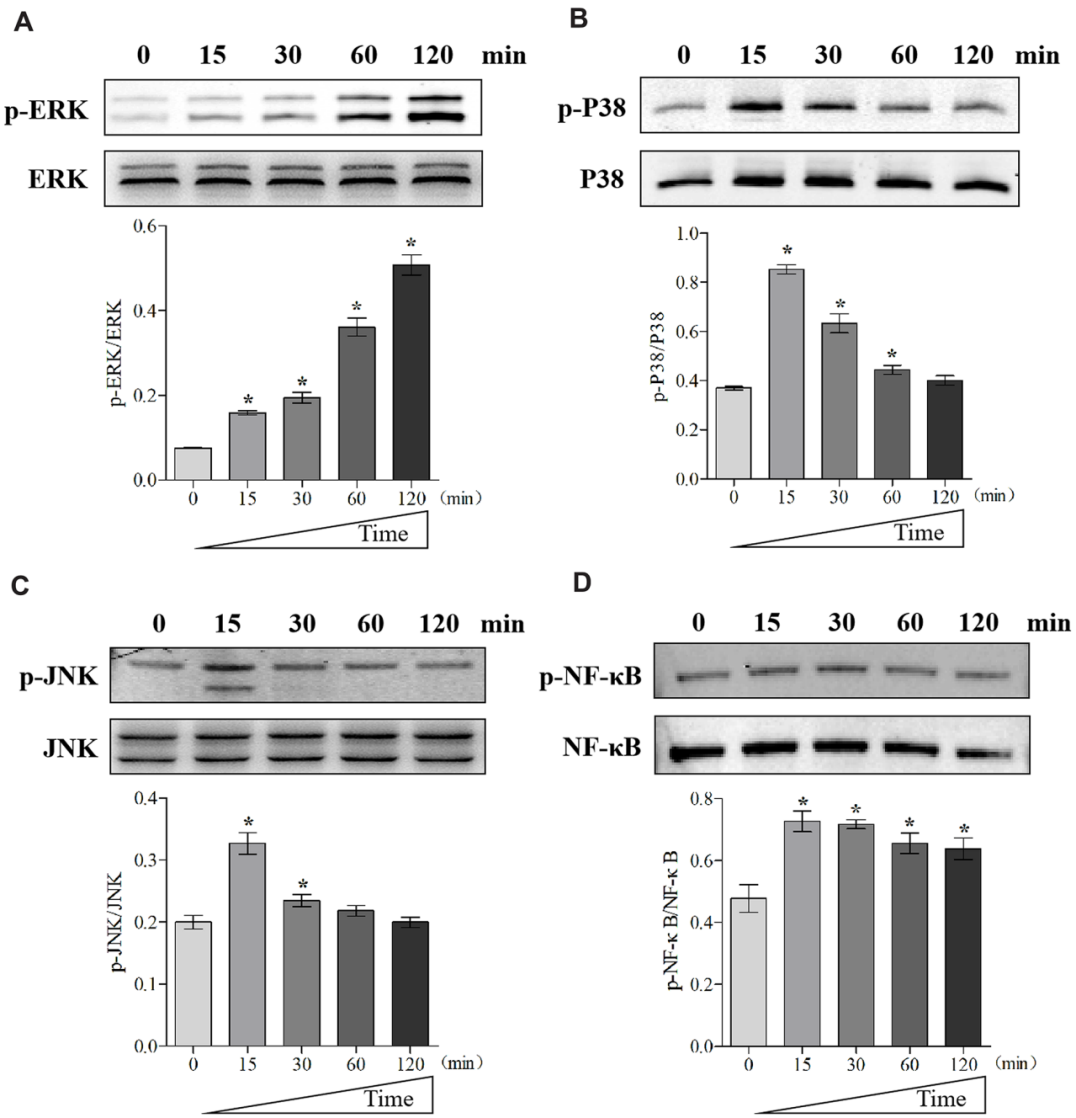
**MIF activated signaling pathways related to inflammation in JFs.** (**A**–**D**) Western blot analysis of MAPK signaling-related proteins p-ERK (**A**), p-P38 (**B**), p-JNK(**C**), and NF-κB signaling-related protein p-NF-κB (**D**) after JFs were treated with 2 μg/mL recombinant MIF for 0, 15, 30, 60, and 120 min. Error bars represent standard deviation. *P <0.05 compared with the 0 min group.

To further unveil the potential mechanism through which MIF regulates JF, we performed transcriptome sequencing analysis on JFs treated with 2 μg/mL MIF for 24 and 48 h. A total of 515 and 1251 DEGs were identified at each time point (Figure 7A, 7B). We further integrated 267 DEGs at the two time points (Figure 7C), of which 118 DEGs were enriched in cellular pathways. KEGG pathway analysis revealed that these DEGs were mainly enriched in functional pathways related to inflammation, immune regulation, and lipid metabolism, among which the MAPK and nuclear factor-κB (NF-κB) pathways were significantly enriched (Figure 7D–7F). Consistent with transcriptome analysis, we found that phosphorylation of MAPK and NF-κB was quickly induced 15 minutes after stimulation of JFs with 2 μg/mL MIF (Figure 8A–8D). Of note, ERK and NF-κB remained continuously activated for 120 min (Figure 8A, 8D), indicating that they likely play a major regulatory role in MIF-mediated JF events.

### MIF induced JF inflammation through CD74 receptor

**Figure 9:**
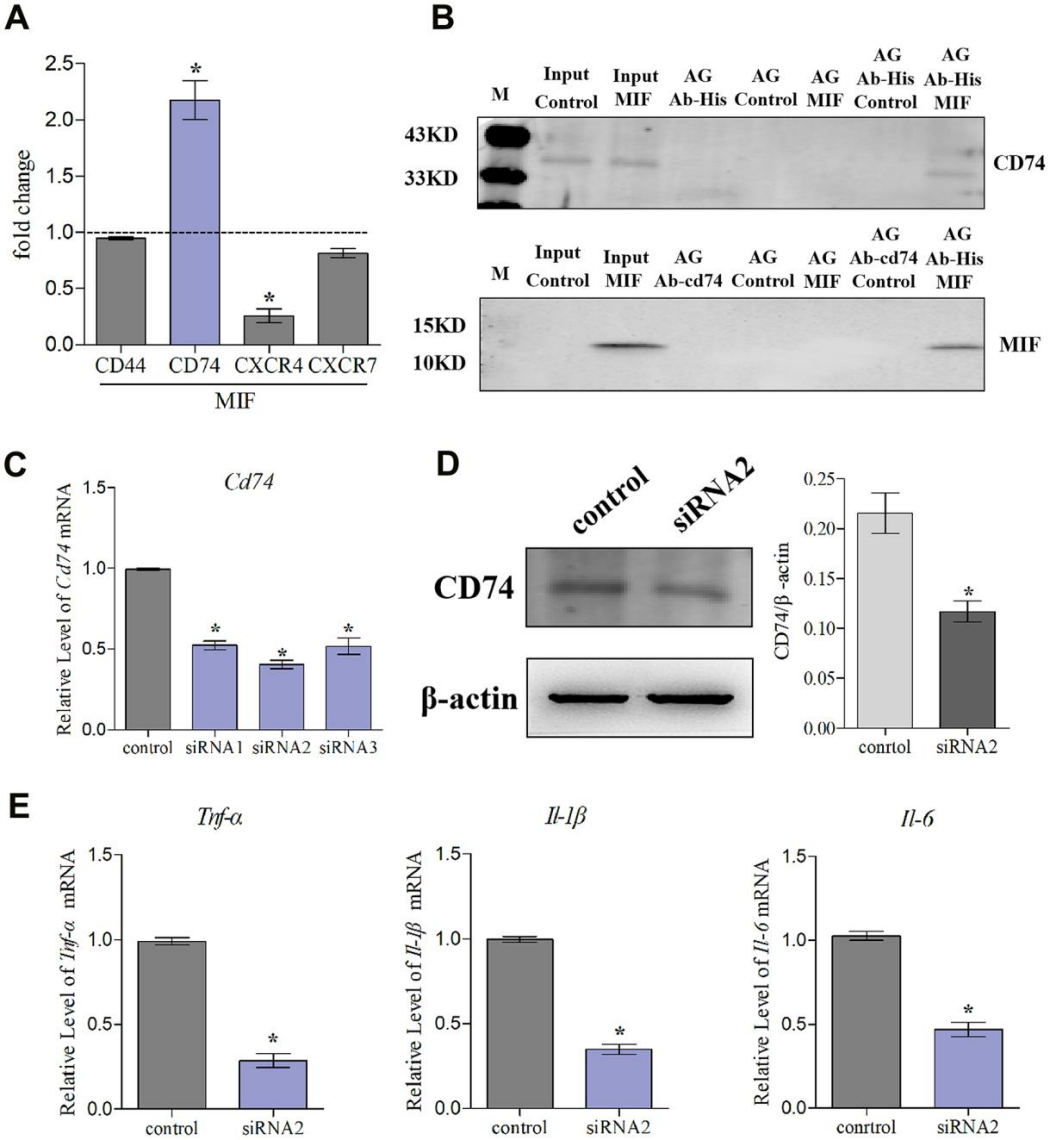
**Knockdown of CD74 affected MIF-induced inflammatory responses in JFs.** (**A**) qRT-PCR analysis of CD44, CXCR4, CXCR7, and CD74 in JFs following treatment with 2 μg/mL recombinant MIF for 24 h. (**B**) Immunoprecipitation was used to determine the interaction between MIF and CD74. (**C**) Knockdown efficiency of CD74 siRNAs were tested using qRT-PCR after transfection for 48 h and siRNA2 was chosen for subsequent experiments. (**D**) Western blot analysis of CD74 following siRNA2 knockdown of CD74 for 48 h. siRNA (control) with the same nucleotide composition as siRNA2 but lacking sequence homology to the CD74 was designed as a negative control. (**E**) Expression of *Tnf-α*, *Il-1β,* and *Il-6* was assessed via qRT-PCR following treatment of JFs with siRNA2 or control for 48 h and stimulation with 2 μg/mL recombinant MIF for 24 h. Error bars represent standard deviation. *P <0.05 compared with the control group.

MIF has been shown to activate intracellular signal transduction by binding to CD74 receptor, which forms a receptor complex with CD44, CXCR2, CXCR4, or CXCR7 [20, 24, 25]. Our results revealed that CD74 was significantly induced by MIF, whereas CD44 was unchanged, CXCR4/7 was decreased, and CXCR2 was not detected in JFs (Figure 9A). Subsequently, Co-IP was performed to confirm the MIF-CD74 interaction. As shown in Figure 9B, CD74 was present in MIF-associated complexes when immunoprecipitation was performed using an anti-MIF antibody. MIF was also observed in CD74-associated complexes. To determine whether this interaction can initiate JF inflammation, we synthesized and evaluated three specific siRNAs targeting CD74 [29], and selected siRNA2 with the highest interference efficiency for subsequent experiments (Figure 9C, 9D). qRT-PCR revealed that CD74 knockdown significantly reduced MIF-induced inflammatory cytokine production (Figure 9E). These data indicated that MIF-induced JF inflammation was dependent on the CD74 receptor.

### Identification of inflammation-related factors downstream of MIF

**Figure 10:**
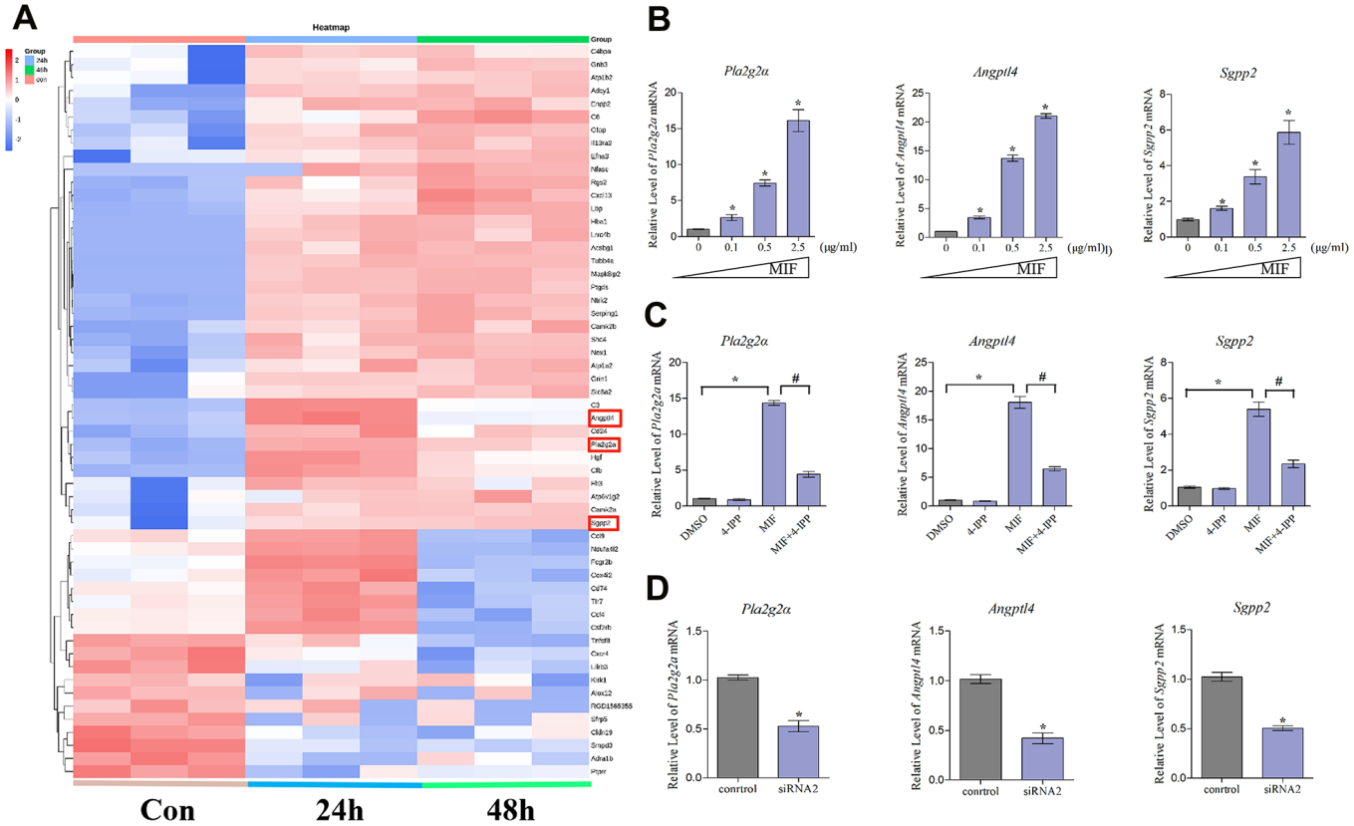
**Expression profiling and inflammation-related factors identification of integrated DEGs analysis.** (**A**) Heat map of integrated DEGs in the top 30 most significantly enriched groups. Red represents upregulation. Blue represents downregulation. (**B**–**D**) qRT-PCR analysis of the expression of *Pla2g2a*, *Angptl4*, and *Sgpp2* in JFs after treatment with 0–2.5 μg/mL recombinant MIF for 24 h (**B**), treatment with 2 μg/mL MIF combined with 50 μ5M 4-IPP for 24 h (**C**), or treatment with siRNA2 for 48 h, followed by stimulation with 2 μg/mL MIF for 24 h (**D**). Error bars represent standard deviation. *P <0.05 compared with the 0 μg/mL or control group.

To identify inflammation-related factors downstream of MIF, we further analyzed 118 integrated DEGs. As shown in the heat map, 57 DEGs that were contained in the top 30 most significantly enriched pathways exhibited dynamic changes after MIF treatment (Figure 10A). Increasing evidence has shown that fibroblast lipid metabolism disorders can induce various inflammatory and fibrotic diseases [9, 37]. Our analysis of the 57 DEGs revealed that phospholipase A2 group IIA (Pla2g2a), angiopoietin-like 4 (Angptl4) and sphingosine-1-phosphate phosphatase 2 (Sgpp2) were prominent inflammatory regulators related to lipid metabolism [17, 38, 39]. Consistent with transcriptome analysis, MIF significantly promoted the expression of the above genes in JFs (Figure 10B). Addition of 4-IPP to the cultures (Figure 10C) or knockdown of CD74 (Figure 10D) effectively blocked the effects of MIF, indicating that the lipid metabolism-related molecules downstream of the MIF/CD74 axis were potentially implicated in mediating the JF inflammation.

## DISCUSSION

PTJC is a major musculoskeletal disease caused by trauma or surgery and is characterized by the loss of passive ROM of diarthrodial joints, resulting in tissue degeneration and deformity [1, 2, 40]. The posterior joint capsule is the key anatomic factor for knee contracture, and its fibrosis causes irreversible loss of joint motion [41–43]. Joint capsule fibrosis is a complex pathological process, including inflammation, collagen fiber hyperplasia, MMPs production, and other factors [4–7, 43]. Considering that inflammation is the pathogenic basis and early event of joint capsule fibrosis [7, 35], we hypothesized that intervention of excessive inflammation can prevent or alleviate PTJC from the source.

Traditionally, inflammation is believed to be driven by inflammatory and immune cells, whereas fibroblasts are involved in the regulation of the ECM composition [42–44]. However, an inflammatory cascade forms as the result of close coordination between stromal cells and inflammatory cells [8–10]. Fibroblasts are known to transform acute inflammation into chronic persistent inflammation in other diseases [15, 45]. Based on these, we speculated that fibroblasts may also play an important role in inflammation and immune regulation during joint capsule fibrosis. Therefore, the work should focus on delineating the key regulatory molecules involved in the activation of JF inflammation next.

MIF plays a key role in inflammation and fibrosis-mediated diseases [17–20]. However, its role in PTJC had not been previously reported. Herein, we observed that MIF was linked to joint capsule inflammation and fibrosis through interactions with JFs. Secreted MIF attacks the JFs through paracrine and autocrine actions [46, 47]. We detected a significant increase in TNF-α, IL-1β, and IL-6 levels following MIF treatment. Notably, the amount of IL-1β secreted into culture supernatant was extremely low, whereas previous studies reported that MIF promoted the secretion of IL-1β in macrophages and dendritic cells [27]. This suggests an unknown regulatory mechanism of IL-1β secretion in JFs, which may depend on the cell type MIF acts on. MIF is also known to produce MMPs which degenerate collagen fiber in inflammatory joint. For example, MIF is reported to induce rheumatoid arthritis synovial fibroblast MMPs production [48, 49]. In our study, we found similar results that MIF promoted MMPs (*Mmp-1*, *Mmp-13*) and collagen (*Col1a1*, *Col3a1*) production in JFs. It seems to be contradictory between MMP production and collagen fiber hyperplasia. Although the original function of MMPs are to regulate collagen fiber degradation. However, they have other properties, including regulation of noncollagenous matrix proteins and enzymes, growth factors, and cellular chemotaxis, proliferation, and apoptosis [50]. For example, during myocardial fibrosis, myofibroblasts activate inflammatory cells to adhere to the endothelium, to release MMP-9 allowing degradation of the basal membrane and subsequent transendothelial migration, and to induce chemokine expression, attracting other inflammatory cells, eventually exacerbate inflammation [8]. It is the other functions (noncollagenous) of MMPs that may contribute to the contracture process.

Fibroblast proliferation is one of the main characteristics of fibrous diseases [34, 35]. Our results were consistent with previous observations that MIF expression increases and promotes fibroblast proliferation during chronic inflammatory diseases [18–21]. Furthermore, we found that MIF effectively promoted JF migration. The fibrotic joint capsule thickening and disorder may be caused by MIF-dependent cell proliferation and migration. To unveil the regulatory mechanism, we performed transcriptome analysis on JFs treated with MIF. KEGG pathway analysis identified that the MAPK and NF-κB signaling pathways were significantly enriched. MAPK is the main intracellular signaling cascade involved in inflammation. MIF can rapidly activate MAPK, leading to the translocation of NF-κB into the nucleus and affecting cell function by regulating gene transcription [17, 26, 36]. Herein, we observed sustained ERK/NF-κB phosphorylation in comparison with P38 and JNK. Considering that inhibition of ERK can alleviate the formation of joint adhesions and joint capsule fibrosis [5, 51, 52], we believe that the ERK/NF-κB pathway is likely to play a major regulatory role in MIF-mediated JF events. In addition, MIF’s high-affinity receptor CD74 was necessary for the activation of MAPK/NF-κB and inflammatory cytokine production in JFs.

Inflammation is regulated by complex mechanisms involving the immune system, renin-angiotensin-aldosterone system, and lipid metabolism [35]. Accumulating evidence has shown that fibroblast lipid metabolism disorders can induce various inflammatory and fibrotic diseases [8, 9, 35]. Our previous studies confirmed that the MIF/CD74 axis affects the inflammatory microenvironment by regulating phospholipid and cholesterol metabolism after spinal cord injury [30, 53]. Interestingly, MIF promotes cell phospholipid metabolism to antagonize the anti-inflammatory effects of glucocorticoids and NSAIDs [17, 18, 30], which may be one of the important reasons why PTJC treatment and prevention remains a challenge. We further analyzed the sequencing results and identified lipid metabolism- and inflammation-related factors downstream of the MIF/CD74 axis, specifically Pla2g2a, Angptl4, and Sgpp2, which may be involved in mediating JF inflammation. Pla2g2a is involved in the regulation of innate/adaptive immunity and is induced by inflammatory cytokines, such as IL-1β and TNF-α [17, 18, 54]. It is closely related to the occurrence of pulmonary fibrosis in humans and rats [55]. Angptl4 is a secretory protein involved in the regulation of angiogenesis, cell differentiation, lipid metabolism, and inflammation [38, 56, 57], which are also closely related to the development of joint capsule fibrosis. Lack of Angptl4 in adipose tissue enhances the clearance of proatherogenic lipoproteins, attenuates inflammation, and reduces atherosclerosis [58]. Sgpp2 can be induced during inflammatory responses [39], and its abnormal expression enhances B cell- and macrophage-mediated inflammation in patients and mice with systemic lupus erythematosus [59]. But its role in fibrosis has not been reported. Follow-up studies should elucidate the regulatory mechanisms of the transcriptional synthesis of these factors, as well as identify the underlying mechanisms through which they affect JF inflammation.

## CONCLUSIONS

MIF acted as a core regulator of JF inflammation. The MIF/CD74 axis promoted JF proliferation, migration, and inflammation, and activation of the MAPK/NF-κB pathway was necessary for required for MIF’s effects. Lipid metabolism-related factors Pla2g2a, Angptl4, and Sgpp2 were identified as potentially implicated in mediating JF inflammation downstream of the MIF/CD74 axis. Our findings identify novel targets for the control of joint capsule inflammation and fibrosis in PTJC.

## MATERIALS AND METHODS

### Animals

Adult male Sprague Dawley (SD) rats (180–220 g) were purchased from Shanghai SIPPR-Bk Lab Animal Co., Ltd. and housed in specific-pathogen-free laboratory animal facilities of Shanghai Ninth People's Hospital. All animal experiments complied with the National Institutes of Health Guide for the Care and Use of Laboratory Animals and were reviewed and approved by the Institution of Animal Care and Use Committee (IACUC) of Shanghai Ninth People’s Hospital. All rats were housed in individual cages at room temperature (23 ± 1° C) and 12 h light/dark cycle with free access to food and water.

### Establishment of rat PTJC model

The PTJC model was established as previously described [5, 51]. Briefly, rats were anaesthetized via intraperitoneal injection of sodium pentobarbital (50 mg/kg). Their fur was shaved from the right knee, and disinfected the skin. A 15-mm midline skin incision was made before performing a lateral parapatellar arthrotomy. The patella was reflected medially to expose femoral condyles. Two 1.5-mm cortical windows were made from non-articulating cartilaginous regions of the medial and lateral femoral condyles using a 1.5-mm drill bit. The anterior/posterior cruciate ligament was sequentially incised, and the knee was hyperextended to −45° C to disrupt the posterior capsule. The knee was immobilized at 135° C of flexion with a 0.5-mm steel wire. Muscles and skin were sutured with silk threads after patellofemoral joint reduction. For drug delivery, 10 μL of 100 mM 4-iodo-6-phenylpyrimidine (4-IPP) or vehicle was injected into the joint cavity. After surgery, rats were allowed unrestricted daily activity in cages. Rats were euthanised at day 0, 1, 3, 7, and 14, removed the internal fixation, and measured knee extension ROM within 15 min of euthanasia. Posterior joint capsules were collected for subsequent analysis.

### Primary JF culture and identification

SD rats (male, 4-week-old) were euthanized via anesthesia overdose, posterior joint capsules were washed with Dulbecco's minimum essential medium (DMEM). Sub-sectioned tissues were placed in culture plates containing DMEM supplemented with 10% FBS, 1% penicillin/streptomycin in a 5% CO_2_ incubator at 37° C [60]. After 3–5 days, fibroblasts started to migrate from the sub-sections. The tissues were removed when the culture reached 90% confluence. The medium was changed every couple of days. Primary JFs were identified via immunofluorescence staining for vimentin (Abcam) before use in subsequent experiments.

### Western blot

The samples of the posterior joint capsule following injury or JFs treated with various stimuli were lysed in RIPA buffer. The BCA Protein Assay Kit (Beyotime) was used to measure protein concentrations. We performed western blotting as previously described [30], and the following primary antibodies were used: β-actin (Proteintech); MIF, Collagen I/II/III, PDGF, TGF-β1 (Abcam); CD74 (Biorbyt); p-ERK/ERK, p-P38/P38, p-JNK/JNK, and p- NF-κB/NF-κB (CST). Secondary antibodies included goat anti-rabbit IgG or goat anti-mouse IgG (Invitrogen). The relative intensities of bands were normalized to β-actin.

### Quantitative RT-PCR

RNA from JFs treated with TNF-α (PeproTech), MIF (ProSpec), 4-IPP (TOCRIS), or CD74-siRNA (Ribobio) was extracted using TRIzol (Invitrogen) according to the manufacturer's instructions. Next, reverse transcription of total RNA was carried out using the Omniscript Reverse Transcription Kit (QIAGEN), and qRT-PCR was carried out using SYBR® Premix Ex Taq™ (Takara Bio) on a real-time PCR system (Applied Biosystems). We normalized gene expression to *Gapdh*. Primers used for qRT-PCR are shown in Table 1 and Supplementary Table 1.

**Table 1:** Primers used in qRT-PCR.

**Gene**	**Forward primers (from 5ʹ to 3ʹ)**	**Reverse primers (from 5ʹ to 3ʹ)**
***Gapdh***	ACAGCAACAGGGTGGTGGAC	TTTGAGGGTGCAGCGAACTT
***Tnf-α***	GCGTGTTCATCCGTTCTCTACC	TACTTCAGCGTCTCGTGTGTTTCT
***Il-1β***	GCTGTCTGACCCATGTGAGCTG	ATTTTGTCGTTGCTTGTCTCTCCTT
***Il-6***	AGTTGCCTTCTTGGGACTGATGT	GGTCTGTTGTGGGTGGTATCCTC
***Mif***	CTTGGGTCACACCGCACTTA	TCGCTCGTGCCACTAAAAGT
***Cd44***	GCAACTACAGCCTTGATGACTA	ATGACTCTTGGACTCTGATGGT
***Cd74***	CATCGGGCTCACAGGTTTGG	CTGGTGGCTCTGCTCTTGGC
***Cxcr2***	CGTCCACGCCACAAGTA	ACGGTAGAAGGGTTTGCC
***Cxcr4***	AAGCAAGGATGTGAGTTCG	AAGGCGTAGAGGATGGG
***Cxcr7***	TCACCTACTTCACCAGCACC	ACATGGCTCTGGCGAGCAGG
***Pla2g2a***	GCTTCTACGGTTGCCATT	GAGTCACACAGCACCAATCT
***Angptl4***	TGACCGACTGGAGATAGGG	GTGAGCTGTGCCTTGGAA
***Snpp2***	CATCGCCCTGACCTACC	TGTAGCACAAGAGGAACGG

### Enzyme-linked immunosorbent assay (ELISA)

JFs were treated with MIF or TNF-α, and cell supernatants and lysates were harvested and centrifuged at 12,000 × g for 15 min. TNF-α, IL-1β, IL-6, and MIF concentrations were assessed using the appropriate ELISA kits (MULTI SCIENCES) according to the manufacturer's instructions.

### Co-immunoprecipitation (Co-IP)

Cell lysates were harvested after treatment of JFs with MIF for 24 h. Total cell lysates (500 μg) were precleared with protein A plus G-Sepharose before incubation with specific antibodies at 4° C, followed by addition of protein A plus G-Sepharose. After several washes, samples were boiled and analyzed via immunoblotting using an anti-MIF or anti-CD74 antibody.

### Cell proliferation assay

JFs were plated at a density of 1 × 10^5^ cells/mL in 96-well plates. EdU was applied to cultures after treatment with 0–2.5 μg/mL MIF or 2 μg/mL MIF in combination with 50 μM 4-IPP for 24 h, and cells were incubated for another 2 h. Cells were then assayed using the Cell-Light EdU DNA Cell Proliferation Kit (Ribobio) according to the manufacturer’s instructions. Analysis of cell proliferation was performed using images of randomly selected fields obtained with a fluorescence microscope (Leica).

### Transwell migration assay

JFs (2 × 10^4^) were transferred to the top Transwell chambers (Costar; 6.5-mm diameter, 8 μm pores) following pre-treatment with 1 μg/mL mitomycin C. 500 μl complete medium containing 0–2.5 μg/mL MIF or 2 μg/ml MIF in combination with 50 μM 4-IPP was added to appropriate lower chambers of a 24-well plate. After migration for 24 h, the upper surface of each membrane was cleaned with a cotton swab. Cells on the bottom surface were fixed with 4% paraformaldehyde, followed by staining with 0.2% crystal violet (Sigma). Analysis of cell migration was performed by counting the number of cells in images of randomly selected fields obtained using an inverted microscope (Leica).

### Immunofluorescence and histological observation

JFs treated with TNF-α were fixed in 4% paraformaldehyde and incubated with anti-MIF (Abcam) antibodies. Fluorescent secondary antibodies were used to visualize bound targets. Cells were then stained with 4′,6-diamidino-2-phenylindole (DAPI; Sigma) and phalloidin (Abcam). Knee joint samples were collected, decalcified in 10% EDTA, and embedded in paraffin. Knee joint specimens were sagittally sectioned and stained with hematoxylin-eosin (HE) and Masson stain. For immunohistochemical analysis, sections were incubated with antibodies against MIF, vimentin, and CD68 (Abcam) overnight. On the next day, sections were incubated with the appropriate secondary antibody and DAPI. All images were captured using a Zeiss LSM710 confocal microscope.

### Transcriptome sequencing and bioinformatics analysis

Total RNA of JFs following treatment with MIF for 24 h and 48 h was extracted using the mirVana miRNA Isolation Kit (Ambion) and selected by RNA Purification Beads (Illumina) for library construction and RNA-seq analysis. The library was constructed by using the Illumina TruSeq RNA sample Prep Kit v2 and sequenced using the Illumina HiSeq 2000. High-quality reads that passed the Illumina quality filters were used for sequencing analysis. Sequencing outcomes were normalized with Reads Per Kilobase per Million mapped reads. Differentially expressed genes (DEGs) were identified according to the criteria of fold change >2 and P <0.05 in comparison with the control. Gene functions were annotated via Blastx against the NCBI databases or AGRIS database with an E-value threshold of 10^−5^. Gene ontology (GO) classification was obtained by WEGO via GO id annotated by a Perl and R program. Kyoto Encyclopedia of Genes and Genomes (KEGG) pathways analysis was performed using the KEGG Automatic Annotation Server. For all heatmaps, genes were clustered via Jensen-Shannon divergence.

### Statistical analysis

All results were expressed as mean ± standard deviation after analysis using the SPSS 22.0 statistical software (SPSS Inc, Chicago, IL, USA). Parametric data were analyzed via Student's t-test or one-way analysis of variance (ANOVA) followed by Tukey's post-hoc analysis for comparison between two groups. When necessary, log or square root transformation was applied to correct skewed distributions to satisfy the assumptions of parametric tests. Nonparametric data were analyzed with the Mann-Whitney U test or, for multiple comparisons, the Kruskal-Wallis test followed by the Dunn's test. Dose-response curves and repeated measures were assessed by two-way ANOVA and, if different treatments were applied, analysis of covariance followed by Tukey for multiple comparisons. P <0.05 was considered statistically significant.

## Supplementary Material

Supplementary Figure 1

Supplementary Table 1
