# Supplementary material for: Macrophage migration inhibitory factor activates the inflammatory response in joint capsule fibroblasts following post-traumatic joint contracture
Source: Aging (Albany NY). 2021 Feb 17;13(4):5804–23. doi: 10.18632/aging.202505 (PMC7950233; doi:10.18632/aging.202505)
Supplement: Supplementary Figure 1 [file aging-13-202505-s001.pdf]

SUPPLEMENTARY FIGURE

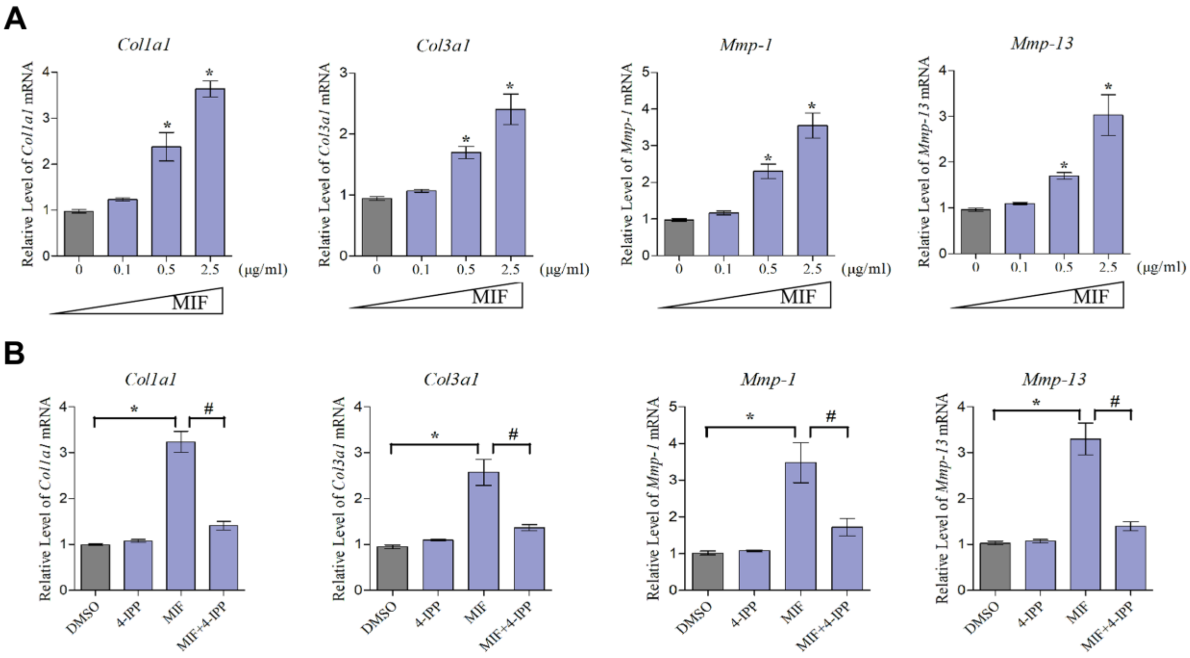

Supplementary Figure 1. MIF promoted MMPs and collagen production in JFs. (**A**) Expression of *Col1a1*, *Col3a1*, *Mmp-1*, and *Mmp-13* was assessed via qRT-PCR following JFs treatment with 0–2.5 μg/mL recombinant MIF for 24 h. (**B**) JFs were treated with 2 μg/mL recombinant MIF combined with 50 μM 4-IPP for 24 h, qRT-PCR evaluated *Col1a1*, *Col3a1*, *Mmp-1*, and *Mmp-13* expression. Error bars represent standard deviation. *P <0.05 compared with the 0 μg/mL or DMSO group. #P <0.05 compared with the MIF group.
