# Supplementary material for: Macrophage migration inhibitory factor activates the inflammatory response in joint capsule fibroblasts following post-traumatic joint contracture
Source: Aging (Albany NY). 2021 Feb 17;13(4):5804–23. doi: 10.18632/aging.202505 (PMC7950233; doi:10.18632/aging.202505)
Supplement: Supplementary Table 1 [file aging-13-202505-s002.pdf]

SUPPLEMENTARY TABLE

Supplementary Table 1. Primers used in qRT-PCR.

Gene	Forward primers (from 5′ to 3′)	Reverse primers (from 5′ to 3′)
Gapdh	ACAGCAACAGGGTGGTGGAC	TTTGAGGGTGCAGCGAACTT
Col1a1	TGTATCACCAGACGCAGAAGT	ACCAGGAGGACCAGGAAGT
Col3a1	ACTGGTGAATGGAGCAAGACA	AAGCAAGAGGAACACATGGATG
Mmp-1	GCCATTACTCACAACAATCCTCG	AACACAATATCACCTTCCTCCTCAA
Mmp-13	TGCAGTCTTTCTTTGGCTTAG	CTCCACATCTTGGTTTTCTCAT
